# The Sydenham Society, with a Prospectus, and a List of the Provisional Officers

**Published:** 1843-04

**Authors:** 


### PART FOURTH.

### Medical Intelligence.

---

### THE SYDENHAM SOCIETY.

[The following prospectus, which we transcribe at length, gives so clear an account of the nature and objects of the new Society, that we have scarcely anything to add to commend it to the notice of our readers. None but those who, like the editors of critical Journals, are called on to examine all publications that issue from the medical Press, can be fully aware of the extent to which ignorance of the medical literature of past times—even of that immediately preceding our own time—prevails among the members of our profession. It is lamentable to think to what an extent the weekly journals constitute the habitual reading of a large portion of medical men, than which nothing can be imagined less likely to advance sound knowledge, or improve good taste. We have some hope that the Works to be issued from the Sydenham Press, and which must, in some degree, compel the attention of the profession to them, may awaken a relish for a higher and healthier literature.]

*Prospectus.*—The Sydenham Society has been founded for the purpose of meeting certain acknowledged deficiencies in the diffusion of medical literature, which are not likely to be supplied by the efforts of individuals. It will carry this object into effect by distributing among its members—

1. Reprints of standard English medical works, which are rare and expensive.
2. Miscellaneous Selections from the ancient and from the earlier modern authors, reprinted or translated.
3. Digests of the most important matters contained in old and voluminous authors, British and foreign, with occasional biographical and bibliographical notices.
4. Translations of the Greek and Latin medical authors, and of works in the Arabic and other Eastern languages, accompanied, when it is thought desirable, by the original text.
5. Translations of recent foreign works of merit.
6. Original works of great merit, which might be very valuable as books of reference, but which would not otherwise be published, from not being likely to have a remunerating sale,—such as classified Bibliographies, and alphabetical Indexes to periodical publications and other valuable voluminous works.

The Society will consist of an unlimited number of members.

The subscription constituting a member is one Guinea annually, for which he will be entitled to a copy of every work printed by the Society, during the time of his subscription.

The subscriptions are to be paid in advance; and no member is responsible beyond the amount of his subscription.

All works published by the Society will be selected by the Council; and, previous to publication, will be subjected to their supervision.

The Society will not commence its operations until the number of its members amounts to five hundred.

The works of the Society will be printed for members only; on a uniform plan, and with a good legible type.

The Society will be under the direction of a Council of twenty-four members, elected at the annual general meeting from the subscribers at large; and of this number eighteen only will be re-eligible for the following year.

The President and Vice-Presidents will also be elected annually.

As the expense of management will be very small, nearly the whole of the funds subscribed will be devoted to the publications; and as the proportionate cost of producing books decreases as the number of copies increases, it is anticipated that, when the Society is fully organized, the annual supply of works to members will be considerable.

The great success that has attended other Societies, established on similar principles, and with like objects,—as the Camden, the Parker, the Percy, &c.,* leaves no room for doubt as to the eventual prosperity of the Sydenham Society. It would indeed be strange, if Medicine, which boasts of a literature more extensive than that of any other art or science, and of cultivators as numerous, zealous, and learned, as any other department of human knowledge, should fail in attaining an end which has been so speedily and so fully accomplished by the societies referred to, and by others embracing even less comprehensive objects.

The Gentlemen named below, as constituting the Provisional Council, have undertaken the organization of the Society, and will continue to act until its constitution and government are finally fixed at a general meeting of the members. They have great satisfaction in being able to lay before their medical brethren the names of the eminent individuals who have consented to act as Provisional President and Vice-Presidents, and who have promised their zealous co-operation in carrying into effect the important objects of the Society.

PROVISIONAL OFFICERS OF THE SYDENHAM SOCIETY.

*President.*

Sir Henry Halford, Bart. G.C.H. M.D. F.R.S. F.A.S. President of the Royal College of Physicians, Physician to the Queen.

*Vice-Presidents.*

John Abercrombie, M.D. F.R.S.E. First Physician to the Queen for Scotland.
W. P. Alison, M.D. F.R.S.E. Professor of Medicine in the University of Edinburgh.
John Blackall, M.D. Physician to the Devon and Exeter Hospital.
Sir Benjamin C. Brodie, Bart. F.R.S. Sergeant-Surgeon to the Queen.
Sir William Burnett, M.D. F.R.S. K.C.H. Inspector-General of Fleets and Hospitals.
John Burns, M.D. F.R.S. Professor of Surgery in the University of Glasgow.
William Frederic Chambers, M.D. F.R.S. K.C.H. Physician to the Queen and to the Queen Dowager.
Sir James Clark, Bart. M.D. F.R.S. Physician to the Queen and to the Prince Albert.
Sir Philip Crampton, Bart. F.R.S. Surgeon-General to the Forces in Ireland.
R. J. Graves, M.D. M.R.I.A. Physician to the Meath Hospital, Dublin.
Sir James M'Grigor, Bart. M.D. F.R.S. L. and Ed. Director-General of the Medical Department of the Army.
John Haviland, M.D. Regius Professor of Physic in the University of Cambridge.
Joseph Hodgson, F.R.S. Surgeon to the General Hospital, Birmingham.
Henry Holland, M.D. F.R.S. Physician Extraordinary to the Queen, and Physician to H.R.H. Prince Albert.
John Kidd, M.D. F.R.S. Regius Professor of Medicine in the University of Oxford.
Benjamin Travers, F.R.S. Surgeon Extraordinary to the Queen, Surgeon in Ordinary to H.R.H. Prince Albert.

*Provisional Council.*

Henry Ancell, Esq.
B. G. Babington, M.D. F.R.S. *Treasurer.*
J. R. Bennett, M.D.
John Clendinning, M.D. F.R.S.
James Copland, M.D. F.R.S.
John Dalrymple, Esq.
Fred. J. Farre, M.D. F.L.S.
John Forbes, M.D. F.R.S.
R. D. Grainger, Esq.
George Gulliver, F.R.S.
W. A. Guy, M.B.
Samuel Lane, Esq.
Sir George Lefevre, M.D. Knt.
John North, Esq. F.L.S.
Jonathan Pereira, M.D. F.R.S.
Benjamin Phillips, F.R.S.
J. Forbes Royle, M.D. F.R.S.
William Sharpey, M.D. F.R.S.
G. S. Sigmond, M.D. F.L.S.
Samuel Solly, F.R.S.
Frederick Tyrrell, Esq.
Alexander Ure, Esq.
Robert Willis, M.D.
Erasmus Wilson, Esq.

*Secretary to the Provisional Council.*—W. G. Burroughs, Esq.

* The present number of subscribers to the Parker Society for publishing theological works, exceeds 7000. The number of members in the Camden is limited to 1200, and that number has long been complete.

